# Minimal residual disease guided radical chemoradiotherapy combined with immunotherapy after neoadjuvant immunochemotherapy followed by adjuvant immunotherapy for esophageal squamous cell cancer (ECMRD-001): a study protocol for a prospective cohort study

**DOI:** 10.3389/fimmu.2023.1330928

**Published:** 2024-01-11

**Authors:** Hesong Wang, Xueyuan Zhang, Xiaohan Zhao, Chunyang Song, Wenzhao Deng, Wenbin Shen

**Affiliations:** Department of Radiation Oncology, Fourth Hospital of Hebei Medical University, Shijiazhuang, Hebei, China

**Keywords:** circulating tumor DNA, ctDNA, minimal residual disease, MRD, esophageal cancer, esophageal carcinoma, neoadjuvant immunochemotherapy, adjuvant immunotherapy

## Abstract

**Introduction:**

For locally advanced, inoperable esophageal cancer, concurrent chemoradiotherapy (CCRT) becomes the norm. Combining immunotherapy with radiotherapy has been shown to improve efficacy. Circulating tumor DNA (ctDNA) is a strong predictor of effectiveness and tumor recurrence and is indicative of minimal residual disease (MRD). Patients with inoperable stage II-III esophageal squamous cell carcinoma (ESCC) are enrolled in the ECMRD-001 trial to evaluate changes in MRD status before and after CCRT combined with immunotherapy and adjuvant immunotherapy following neoadjuvant immunochemotherapy.

**Methods and analysis:**

The ECMRD-001 trial is a prospective cohort study. Eligible patients will receive radical concurrent chemoradiotherapy combined with immunotherapy after neoadjuvant immunochemotherapy, followed by adjuvant immunotherapy for at least one year. Follow-up will be up to three years. MRD-related blood and tissue samples and T-cell immunohistobank related blood and tissue samples collected before, during and after treatment and follow-up will be grouped into sample collection time points. The relationship between MRD status at different time points and treatment efficacy is the primary outcome. Correlation between MRD status and immune microenvironment, radiotherapy dose, and tumor recurrence are the secondary outcomes. Examination of ctDNA mutations is the exploratory outcome.

**Discussion:**

ctDNA-based MRD may be a potential predictive marker for the efficacy and tumor recurrence of inoperable ESCC patients. Elevated ctDNA-MRD may predict tumor recurrence earlier than imaging. ctDNA-based MRD analysis and ctDNA-based MRD guided diagnosis and treatment should be implemented into clinical practice to improve efficacy and reduce tumor recurrence of inoperable stage II-III ESCC.

**Trial registration:**

The ECMRD-001 study has been registered at ClinicalTrials.gov as NCT05952661 (July 19, 2023), https://classic.clinicaltrials.gov/ct2/show/NCT05952661.

## 1 Background

Globally, esophageal cancer is among the most common and fatal malignancies, ranking seventh and sixth in incidence and mortality, respectively (1). Different pathological types of esophageal cancer exhibit a distinct epidemiological distribution. The incidence of esophageal adenocarcinoma is high in Europe and North America. While esophageal squamous cell carcinoma (ESCC) is predominant in East Asia (including China), Eastern Africa, and Southern Europe. In China, the incidence of ESCC is predominantly esophageal squamous carcinoma, with the top 5 incidence rates in the world (2). According to the Global Cancer Statistics 2020 report, approximately 604,000 new cases and 544,000 new ESCC deaths were reported worldwide, with China accounting for >50% of the new diagnoses and deaths (3).

Early symptoms of esophageal cancer are not evident, and surgery is mostly avoided because patients are usually detected in the late stage. In the RTOG-8501 study, concurrent chemoradiotherapy (CCRT) significantly increased local control rates and improved survival compared with radiotherapy alone. CCRT is the standard of care for unresectable, locally advanced esophageal cancer (4). Moreover, in the RTOG-9012 study, CCRT offered no survival benefit at higher doses compared with standard radiation doses, but possibly increased treatment-related mortality (5). Based on the results of several studies, the NCCN guidelines recommended a standard radical radiation dose of 50.0–50.4 Gy for inoperable cT1b-T4aN0-N(+) and cT4b stage esophageal cancer (including esophageal adenocarcinoma and ESCC) and 60–66 Gy for inoperable cervical esophageal cancer (6). Unlike European and American countries, the pathological type of esophageal cancer reported from China is mostly ESCC. Moreover, the screening mechanism used in China is imperfect, with a high proportion of intermediate to advanced-stage ESCC cases observed at first diagnosis and a preference for a radical radiation dose of 60 Gy among physicians. However, even after receiving high doses (≥60 Gy) of radiotherapy with concurrent chemotherapy treatment, esophageal cancer patients have a 2-year overall survival rate of only 35.47% (7), which suggests the requirement for follow-up consolidation therapy.

Cancer cells should normally be recognized by the immune system as foreign cells and removed. However, cancer cells have mechanisms to evade immune checkpoints and shut down the immune response against themselves. Therefore, immune checkpoint inhibitors (ICIs) block the interaction between immunosuppressive molecules and target cancer cells (e.g., PD-L1) and their receptor counterpart immune effector T cells (e.g., PD-1). A lot of evidence is available proving the effectiveness of ICI treatment. The KEYNOTE-181 phase III study established the beneficial role of pablizumab (a PD-1 inhibitor) in the treatment of advanced esophageal cancer (8). Several animal model studies have suggested that combining radiotherapy with ICIs targeting T cells is a good synergistic option for inducing other systemic antitumor immune responses (9, 10). Two phase III clinical studies (NCT03957590, NCT03604991) exploring the efficacy of radiotherapy combined with ICIs against esophageal cancer are underway.

Minimal residual disease (MRD) refers to the small number of cancer cells that remain in the body even after cancer treatment. These residual cancer cells have either not responded to treatment or are resistant to treatment. The number of such cells may be small, and they may trigger no signs or symptoms. However, these cells may cause cancer recurrence. The residual cells are undetectable by traditional methods, such as observing the cells under a microscope and/or tracking abnormal serum protein markers (tumor markers) in the blood.

The MRD concept was first introduced in hematological tumors and was determined to be crucial in assessing treatment efficacy and recurrence risk. Studies have demonstrated that higher MRD loads are associated with a greater recurrence risk and poorer drug efficacy (11). According to different studies, MRD has a good prognostic value in many solid tumors including lung cancer, colorectal cancer, breast cancer, and ESCC (12). A consensus on the detection and clinical application of MRD in lung cancer was finally achieved at the 18th China Lung Cancer Summit. At this summit, leading experts in the clinical, basic, and testing fields of lung cancer conducted in-depth exchanges and discussions on “Research progress and clinical application of microscopic/molecular residual disease in lung cancer” (13). According to this expert consensus, the technical requirements for MRD testing are as follows: (1) Basic technologies for MRD testing, including tumor-informed assays (individualized customization) and tumor agnostic assays (next-generation sequencing [NGS] panels and multi-omics technologies), are currently in the exploratory stage, and prospective studies need to be conducted to determine their sensitivity and specificity. (2) Using NGS technology, the patient’s class I/II gene variants must be covered in the selected multigene panel, with the basic technical criterion being that circulating tumor DNAs (ctDNAs) must be stably detected at an abundance of ≥0.02%. (3) For non-small cell lung cancer involving positive driver genes, the MRD molecular panel should include the driver gene. (4) The MRD assessment report must include cfDNA abundance, ctDNA abundance, and the VAF (variant allele frequency) value of the tested gene.

Some studies have investigated the significance of ctDNA-based MRD surveillance for the early treatment and prognostic assessment of ESCC. A study reported that both the number and frequency of blood ctDNA mutations in patients with esophageal adenocarcinoma increase with the tumor stage and that changes in ctDNA levels can be detected before tumor progression is detected on imaging. Therefore, more aggressive treatment may be planned to improve the prognosis of patients having an increased number or frequency of ctDNAs at earlier stages (14).

To analyze the specificity and role of ctDNA in ESCC, Luo et al. sequenced tumor, paracancer, and normal tissues as well as pre- and postoperative plasma from 11 ESCC patients. They found that the mutation of some loci in postoperative plasma ctDNA was significantly reduced or even cleared compared with that in preoperative plasma. This indicated the feasibility of using ctDNA testing to track the ESCC status and monitor treatment effects (15). Azad T et al. performed cancer personalized profiling by deep sequencing on DNA extracted from the blood of healthy controls and ESCC patients before and after radiotherapy. The results suggested that ctDNA can reflect ESCC-related genetic information and is closely related to ESCC occurrence and progression. ctDNA is expected to be a biomarker reflecting the treatment effect on ESCC and disease prognosis (16).

MRD is currently a hot research topic, and more clinical findings are required to confirm whether MRD can be used as a predictor of outcomes of ESCC patients receiving adjuvant therapy following radical CCRT and adjuvant immunotherapy.

## 2 Methods/design

### 2.1 AIM

The main research aims are to assess changes in the MRD status of patients with inoperable stage II–III ESCC before and after radical CCRT combined with immunotherapy after neoadjuvant immunochemotherapy, followed by adjuvant immunotherapy and its correlation with the efficacy of adjuvant immunotherapy.

The secondary research aims are to explore (1) differences in the efficacy of neoadjuvant immunochemotherapy in patients with MRD-positive versus MRD-negative blood before radical CCRT was applied; (2) differences in the immune microenvironment in patients with different efficacy responses following radical CCRT combined with immunotherapy; (3) differences in the MRD status between treatment with radiation doses of 50 and 60 Gy, and its correlation with patient prognosis; (4) the association between serial changes in the MRD status and the efficacy of adjuvant immunotherapy; and (5) the timing of MRD warning of recurrence in patients ahead of imaging cues.

The exploratory research aim is to explore the ctDNA mutation profiles of ESCC patients undergoing adjuvant immunotherapy.

### 2.2 Study design

This is a prospective cohort study (ECMRD-001 trial). The study is initiated by and will be conducted at the Fourth Hospital of Hebei Medical University. The study has been approved by the ethics committee of the Fourth Hospital of Hebei Medical University (2023KS004) and has been registered at ClinicalTrials.gov (NCT05952661). All patients will have to provide written informed consent before the study begins. The study will be conducted according to the Declaration of Helsinki (64th World Medical Association General Assembly, Fortaleza, Brazil, October 2013). Patients or the general public were not involved in the design, conduct, reporting, or dissemination plans of our research.

### 2.3 Patient selection and recruitment

**Table 1** presents the inclusion and exclusion criteria of the ECMRD-001 trial.

**Table 1:** Inclusion and exclusion criteria of the ECMRD-001 study protocol.

Inclusion criteria	Exclusion criteria
(1) age: 18 - 75 years (2) gender: both sexes, as balanced as possible (3) patients with clinically confirmed TNM 8th stage II-III ESCC by histopathology and are not suitable for surgery (4) patients receive neoadjuvant immunochemotherapy, followed by radical CCRT combined with immunotherapy and finally adjuvant immunotherapy (5) Eastern Cooperative Oncology Group (ECOG) score: 0-1 (6) the functional condition of the organ meets the following requirements- haematological indicators: absolute neutrophil count ≥ 1.5 * 10^9^/L, platelet count ≥ 100 * 10^9^/L, haemoglobin count≥ 9 g/dL; good coagulation: platelet count ≥ 100 x 109/L. Liver: total bilirubin ≤ 2 times the upper limit of normal, ghrelin and ghrelin ≤ 2.5 times the upper limit of normal. Renal: creatinine ≤ 1.5 times the upper limit of normal, or creatinine clearance ≥ 60 mL/min (calculated by the Cockcroft-Gault formula) (7) women of childbearing age must have a urine pregnancy test with a negative result within 7 days prior to starting treatment (8) patients understand and voluntarily sign the informed consent form	(1) patients have been diagnosed or treated for another malignancy within 5 years prior to the start of this study (2) adenocarcinoma, mixed adenosquamous or other pathological types of esophageal cancer (3) any unstable systemic disease, including: active infection, uncontrolled hypertension, unstable angina, angina pectoris starting within the last 3 months, congestive heart failure (≥ New York Heart Association [NYHA] class II), myocardial infarction (6 months prior to enrollment), severe arrhythmia requiring medication, liver, kidney or metabolic disease (4) with known or suspected active autoimmune disease (5) previous treatment with anti-PD-1, anti-PD-L1, anti-PD-L2, anti-CD137 or anti-CTLA-4 antibodies or any other antibodies or drugs that specifically target T-cell co-stimulation or checkpoint pathways (6) known history of testing positive for human immunodeficiency virus (HIV) or known to have acquired immunodeficiency syndrome (AIDS) (7) female patients who are pregnant or breastfeeding (8) other conditions deemed unsuitable for enrolment by the investigator

All patients will be discussed in a multidisciplinary tumor board. The patients will receive a patient information letter and will be included in the study only if they voluntarily sign the informed consent form.

### 2.4 Study procedures

**Figure 1:**
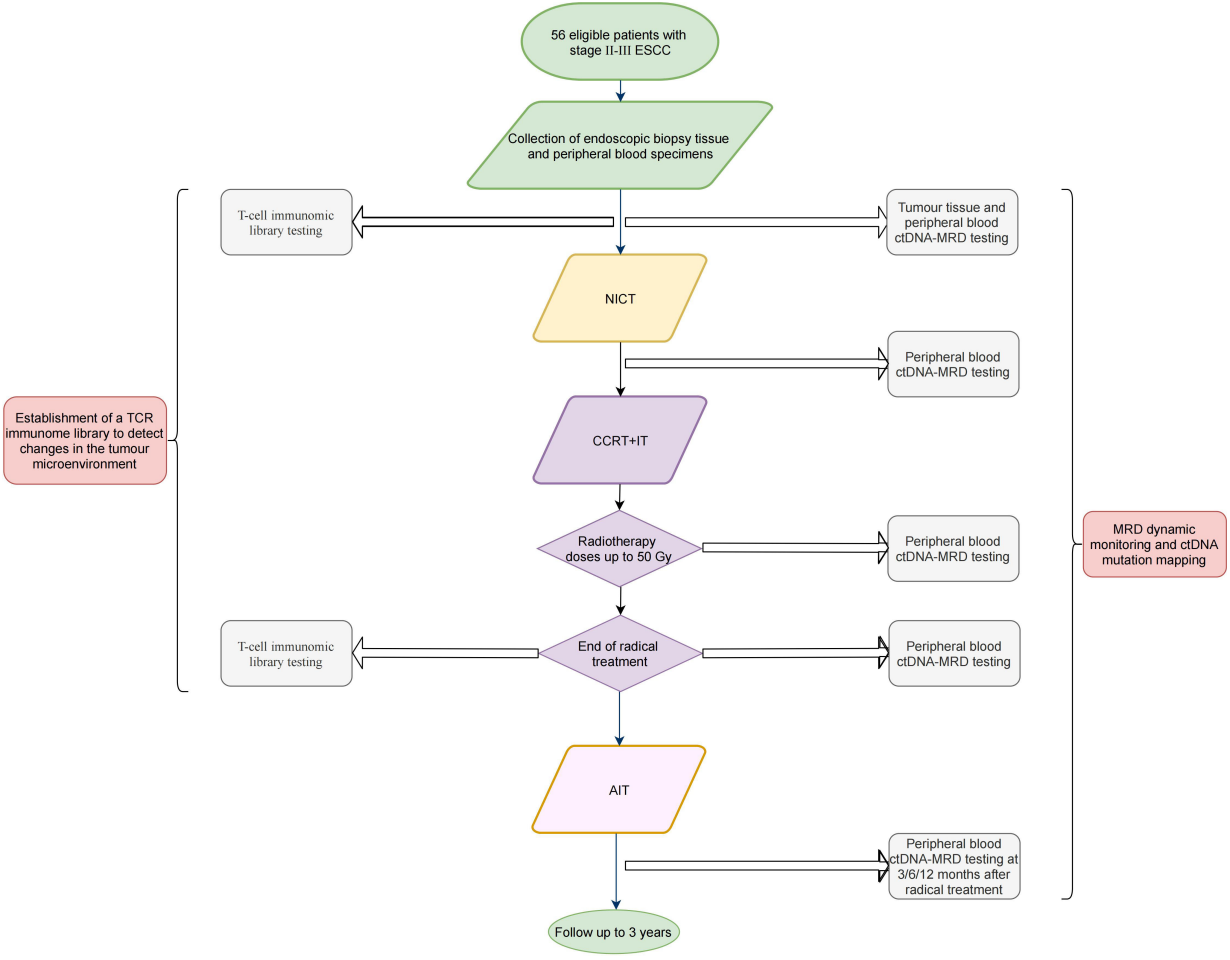
Flowchart of the (ECMRD-001) study. ESCC, esophageal squamous cell carcinoma; ctDNA, circulating tumor DNA; MRD, minimal residual disease; NICT, neoadjuvant immunochemotherapy; CCRT+IT, concurrent chemoradiotherapy combined with immunotherapy; AIT, adjuvant immunotherapy.

**Figure 1** presents the study flowchart.

### 2.5 Neoadjuvant immunochemotherapy

The patients will be intravenously receiving two cycles of albumin-bound paclitaxel at 260 mg/m2 d1 q3w and cisplatin at 75 mg/m2 d1 q3w, with concurrent tislelizumab at 200 mg d1 q3w.

### 2.6 CCRT combined with immunotherapy

The radiotherapy regime to be used is as follows: the gross target volume (GTV) includes the esophageal lesion. The clinical target volume (CTV) is defined as the upper and lower 2.0–3.0 cm and the anterior and posterior 1.0–1.5 cm of the GTV. The planned target volume (PTV) is defined as the upper and lower 1.0–1.5 cm and the anterior and posterior 0.5–0.8 cm of the CTV. Metastatic lymph nodes are defined as GTV-nd, and the uniform 0.5–0.8 cm of the GTV-nd is defined as PTV-nd. All patients will undergo simultaneous integrated boost intensity-modulated radiotherapy, and the prescribed dose is 95% PTV and PTV-nd of 54 Gy, GTV and GTV-nd of 60 Gy, routinely divided, 1.8–2.0 Gy/fraction, 5 times a week.

The patients will receive two cycles of concurrent chemotherapy: albumin-bound paclitaxel at 200 mg i.v. d1,8 q3w and cisplatin at 25 mg/m2 i.v. d1–3 q3w.

The patients will also be administered two cycles of concurrent immunotherapy: tislelizumab at 200 mg i.v. d1 q3w.

### 2.7 Adjuvant immunotherapy

The patients will receive tislelizumab at 200 mg i.v. d1 q3w. The patients will be treated for at least 1 year or until their condition does not progress or they will not tolerate side effects of treatment.

### 2.8 Histological and hematological tests

The specimen collection time points are categorized into six MRD-related blood collections, one MRD-related tissue collection, two T-cell immunohistobank-related blood collections, and one T-cell immunohistobank-related tissue collection before, during, and after the treatment and follow-up.

Before the treatment, the solid tumor specimen of esophageal cancer will be collected from the patients within 5 days before the first cycle of neoadjuvant immunochemotherapy, along with 10 mL*2 tubes of blood for ctDNA-MRD testing and T-cell immunomic library testing.

During the treatment, 10 mL*2 tubes of blood will be collected from the patients 1 day before CCRT combined with immunotherapy at the end of the second cycle of neoadjuvant immunochemotherapy for ctDNA-MRD testing. Then, 10 mL*2 tubes of blood will be collected from the patients for ctDNA-MRD testing when the radiation dose reaches 50 Gy during the CCRT combined with immunotherapy.

Within 3 months after the completion of CCRT combined with immunotherapy, 10 mL*2 tubes of blood will be collected from the patients for ctDNA-MRD testing and T-cell immunobanking.

At the 6- and 12-month follow-ups after the completion of CCRT combined with immunotherapy, 10 mL*2 tubes of blood will be collected from the patients for ctDNA-MRD testing.

### 2.9 Follow-up, efficacy, and safety assessment

Treatment efficacy will be assessed at the end of two cycles of neoadjuvant immunochemotherapy, when a radiation dose of 50 Gy is administered during CCRT combined with immunotherapy, within 3 months of the end of CCRT combined with immunotherapy, at every 2 months during 2 years of adjuvant immunotherapy and follow-up, and at every 3–6 months during the third year of follow-up. The follow-up period is 3 years. The major modalities that will be performed are enhanced CT of the chest and abdomen, barium esophagography, PET-CT and electrogastroscopy (if necessary), MRI of the head, and bone scan. General CT will be performed in patients allergic to contrast agents. The assessment tool that will be used is immune-related response evaluation criteria in solid tumors (irRECIST) for immunotherapy, which will evaluate complete remission (CR), partial remission (PR), stable disease (SD) and progressive disease (PD). The severity of all treatment-related adverse events (trAEs)observed in the patients during and after treatment will be graded according to the National Cancer Institute Common Terminology Criteria for Adverse Events (NCI-CTCAE) version 5.0.

### 2.10 Study endpoints

The primary endpoint is to assess changes in the MRD status of patients with inoperable stage II–III ESCC before and after radical CCRT combined with immunotherapy after neoadjuvant immunochemotherapy, followed by adjuvant immunochemotherapy, and its correlation with the efficacy of adjuvant immunotherapy. In patients with esophageal cancer, previous studies have shown that ctDNA suggests disease recurrence progression in patients on average about 3.5-5.5 months earlier than imaging (16, 17). In addition, several studies have also shown that according to monitoring the dynamic changes of ctDNA can also reflect the patient’s neoadjuvant therapy as well as the efficacy of adjuvant therapy. According to the results of a recent study, 13 patients with esophageal cancer received neoadjuvant chemotherapy, and ctDNA turned negative after neoadjuvant chemotherapy in 3/4 of the patients with pathological remission, and ctDNA positivity after neoadjuvant chemotherapy was significantly correlated with pathological remission (17). Therefore, ctDNA can be used as a potential marker to predict pathological remission and postoperative recurrence in ESCC, and MRD status is a strong predictor of outcome and tumor recurrence in ESCC patients. In this study, we used next-generation sequencing technology in combination with Rdvision 229 gene-immobilized solid tumor panel, a tumor gene detection panel validated in thousands of samples, to detect mutations in tumor tissues and blood samples of eligible esophageal cancer patients at different stages of the study, and to assess the MRD status. MRD test results The MRD test results were determined by negative and positive, with “negative” indicating a low likelihood of residual tumor cell molecules in the patient’s blood, and “positive” indicating that residual tumor cell molecules were detected in the patient’s blood. If ctDNA can detect one or more cancer driver genes or other class I/II gene variants, it will be judged as “positive”; otherwise, it will be “negative”, and the minimum detection limit of gene mutation is 0.02%. Data from several studies have confirmed the predictive value of ctDNA-based MRD testing in the risk of recurrence of esophageal cancer and other solid tumors, as well as MRD as a potential prognostic factor for the efficacy of immune-assisted therapy, which has provided a reference for personalized regimen development of immune-related therapy. However, there is a lack of data support from prospective studies as to whether MRD can be used to guide decision-making for immunoadjuvant therapy following concurrent radiotherapy in patients with ESCC. Therefore, in this study, we propose to use MRD status as a predictive marker for the efficacy of radical radiotherapy and immunoadjuvant therapy in esophageal squamous carcinoma, and to prospectively explore the clinical applicability and value of MRD results based on ctDNA detection to evaluate the efficacy of simultaneous radiotherapy and immunoadjuvant therapy in inoperable esophageal squamous carcinoma patients, in order to screen the patients who can really benefit significantly from immunoadjuvant therapy.

The secondary endpoints are to explore (1) differences in the efficacy of neoadjuvant immunochemotherapy in patients with MRD-positive versus MRD-negative blood before radical CCRT was applied; (2) differences in the immune microenvironment in patients with different efficacy responses following radical CCRT combined with immunotherapy; (3) differences in the MRD status between treatment with radiation doses of 50 and 60 Gy, and its correlation with patient prognosis; (4) association between serial changes in the MRD status and the efficacy of adjuvant immunotherapy; and (5) the timing of MRD warning of recurrence in patients ahead of imaging cues. Firstly, in terms of the immune microenvironment, several studies have shown that the combination of radiotherapy with ICIs targeting T cells may be a good synergistic option for inducing other systemic anti-tumor immune responses, and there have been two phase III clinical studies (NCT03957590, NCT03604991) exploring the therapeutic efficacy of radiotherapy in combination with ICIs in esophageal cancer, and are expected to determine the role of radiotherapy combined with chemotherapy and immunotherapy in esophageal cancer (18, 19). However, the efficacy of immunotherapy varies from patient to patient. Therefore, there is value in assessing patient efficacy and identifying more people who will benefit from immunotherapy, and in using dynamic changes in MRD before and after treatment for immunotherapy efficacy assessment. Secondly, concurrent radiotherapy has become the standard treatment modality for unresectable locally advanced esophageal cancer. However, it has been shown that increasing the dose did not result in a survival benefit and may increase treatment-related mortality compared with the standard radiotherapy dose (5). The NCCN guidelines recommend a standard radical radiotherapy dose of 50.0-50.4 Gy for concurrent chemoradiotherapy of inoperable cT1b-T4aN0-N(+) and cT4b stage esophageal cancers (including adenocarcinomas and squamous cell carcinomas), and a dose of 60-66 Gy for inoperable cervical esophageal cancers. Unlike European and American countries, the pathological type of esophageal cancer in China is mostly squamous cell carcinoma, and the screening mechanism is imperfect, with a high proportion of first diagnosis of intermediate and advanced stages, and the dose of radical radiotherapy is more inclined to be 60 Gy. However, even after experiencing a high dose (≥60 Gy) of radiotherapy with concurrent chemotherapy, the 2-year OS rate of patients with esophageal cancer is still only 35.47% (7). Therefore, MRD can be utilized as an important indicator of how to assess the prognosis of different radiotherapy doses. Finally, in the field of esophageal cancer, some studies have explored the value of ctDNA-based MRD monitoring for early treatment and prognostic assessment of esophageal cancer. In patients with esophageal cancer, previous studies have shown that ctDNA suggests the progression of disease recurrence in patients about 3.5-5.5 months earlier than imaging on average (16, 17). In summary, this study conducted a full managed MRD status test at different stages during the treatment period, and based on the changes in MRD status before and after the treatment, combined with the patient’s efficacy response to immunotherapy, as well as monitoring the patient’s time to MRD positivity and time to imaging progression, correlation analyses were performed to do a correlation study.

The exploratory endpoint is to explore the ctDNA mutation profiles of ESCC patients undergoing adjuvant immunotherapy.

### 2.11 Statistical analysis

#### 2.11.1 Sample size calculation

This single-arm clinical trial will assess the prognostic effect of adjuvant immunotherapy in MRD-negative versus MRD-positive patients with ESCC. This study used PASS software (version 15.05, NCSS, LLC. Kaysville, Utah, USA) to estimate the sample size. And 56 samples were finally used as the sample size for this study. The detailed elaboration and calculation process is placed in **Supplementary Material 1**.

#### 2.11.2 Data analysis

Statistical analysis will be performed using SAS statistical software (version 9.4 or above). Measures will be statistically described by the number of cases, mean, standard deviation, median, maximum, and minimum values; count or rank data will be expressed as frequency; and event time class endpoints will be summarized using Kaplan–Meier estimates.

## 3 Discussion

To our knowledge, the ECMRD-001 study is the first clinical trial to investigate ctDNA-based MRD guided strategies in stage II-III ESCC patients who receive radical chemoradiotherapy combined with immunotherapy after neoadjuvant immunochemotherapy followed by adjuvant immunotherapy.

To our knowledge, the ECMRD-001 study is the first clinical trial to investigate ctDNA-based MRD guided strategies in stage II-III ESCC patients who receive radical chemoradiotherapy combined with immunotherapy after neoadjuvant immunochemotherapy followed by adjuvant immunotherapy.

Numerous studies have demonstrated that ctDNA can be used for assessing treatment efficacy and recurrence risk in ESCC patients. Morimoto et al. (17) observed that longitudinal monitoring of ctDNA during the clinical course of patients with locally advanced ESCC reflects changes in tumor load. Based on these results, ctDNA was considered a potential predictive marker for the efficacy of preoperative neoadjuvant therapy. Moreover, postoperative ctDNA was strongly associated with postoperative recurrence, which suggests that ctDNA reflects MRD after radical surgery. They found that the ctDNA positivity rate following neoadjuvant therapy exhibited a significant association with pathological remission (remitters, 25%; non-remitters, 100%; p = 0.007), with the sensitivity of ctDNA in predicting remission being 1.00, specificity being 0.75, the false-positive rate being 0.25, the false-negative rate being 0, and accuracy being 0.91. Following surgery, 1-year recurrence-free survival was 90% in ctDNA-negative patients compared with 0% in ctDNA-positive patients (p = 0.0008). In their study, the ESCC patients had elevated ctDNA levels approximately 6 months before imaging suggested recurrence. ctDNA monitoring can accurately assess the post-surgery recurrence risk and guide whether adjuvant therapy should be administered. By contrast, no study has explored the role of ctDNA-based MRD as a guide for improving treatment efficacy and reducing the recurrence risk in patients with inoperable ESCC. The present trial aims to assess changes in the MRD status of patients with inoperable stage II–III ESCC before and after radical CCRT combined with immunotherapy after neoadjuvant immunochemotherapy, followed by adjuvant immunotherapy, and its correlation with the efficacy of adjuvant immunotherapy. This study will also explore the relationship between the radiotherapy dose, MRD status, and treatment efficacy; the early prediction of tumor recurrence on the basis of the MRD status compared to imaging; and ctDNA mutation profiles.

This study design has several advantages. First, this study is the first to apply the MRD concept to ESCC patients treated with neoadjuvant immunochemotherapy, followed by radical CCRT combined with immunotherapy and adjuvant immunotherapy. In this study, MRD changes will be measured dynamically throughout the patient’s treatment period and over 12 months. The MRD level and its changes can direct the adjustment of patients’ treatment plans, predict recurrence or metastasis, and guide the development of individualized treatment plans, etc. Second, whether the radical radiotherapy dose for patients with ESCC should be 50 or 60 Gy is still being debated. In this study, MRD testing and efficacy assessment will be performed at 50 Gy and again at 60 Gy. The study aims to compare MRD levels detected in patients at these two doses and their relationship with patient prognosis. The study also aims to further clarify whether 50 or 60 Gy is more reasonable as the optimal dose of radiotherapy for ESCC patients. Third, applying chemotherapy in combination with immunotherapy as a neoadjuvant treatment for ESCC patients is widely practiced clinically, but sufficient medical evidence is lacking. This study will examine the effectiveness of immunotherapy combined with chemotherapy and its value as an emerging neoadjuvant treatment option by examining the MRD status before and after neoadjuvant treatment and assessing treatment efficacy. Fourth, the field of cancer immunotherapy is rapidly developing, and the mechanisms of resistance to immunotherapy must be understood in-depth so as to investigate strategies to address this problem of resistance. Developing a new combination therapy may take longer, and the vast majority of available treatment combinations require additional studies to determine optimal dosing and timing, safety, and efficacy. Exploring mechanisms underlying resistance to ICIs in detail, implementing appropriate measures to avoid drug resistance, reducing the frequency of drug resistance, and eliminating drug resistance-associated adverse effects will certainly promote the rapid development of tumor immunotherapy and bring enduring survival benefits to tumor patients. Individualized immunotherapy is the way forward for tumor immunotherapy and its breakthrough depends on the continuous development and successful translation of basic research. Because of its advantages, the ctDNA NGS assay is now commonly used for analyzing molecular mechanisms underlying acquired tumor resistance and subsequent drug guidance, but no study has evaluated the usefulness of the ctDNA NGS assay in ESCC. In this study, we will use data from MRD dynamic monitoring to judge treatment resistance in patients. Fifth, in this study, we will construct ctDNA mutation profiles of ESCC patients treated with neoadjuvant immunochemotherapy, followed by radical CCRT. These profiles will be used to characterize genomic changes in ESCC patients before and after treatment and will have significant implications in terms of identifying potential therapeutic targets, improving sensitivity to ESCC, and avoiding side effects of ESCC treatment.

The study design is also associated with challenges and limitations. The current sample size for MRD testing for esophageal cancer is small, the current proposed enrolment of 56 patients may not be able to meet the final analysis needs. Therefore, this limitation can be solved by considering appropriate expansion and adjustment of the number of patients to be enrolled based on the results of the phase analysis. In addition, the study was based on MRD analysis of ctDNA as a predictive marker, however, there are some limitations regarding the detection technology of MRD. Currently, the clinical significance of MRD has been supported by the findings of many studies, but there are no uniform standards for the detection technology platform of MRD, the definition of MRD positivity, and the specific optimal time point for monitoring. Firstly, for MRD detection technology platforms, the ctDNA-MRD assays for solid tumors are now divided into two main categories: sequencing of primary tumor tissues to identify patient-specific genomic variants, followed by designing primers to customize the panel for personalized ctDNA analysis, known as ‘Tumor-informed assays’. assays”; without the need for primary tumor tissue, relying only on a fixed panel of pre-selected primers/probes designed to correlate with the type of cancer (usually multi-omics) is called “Tumor-agnostic assays”. Currently, tumor-informed assays have the advantage of being able to perform ultra-high depth sequencing and higher sensitivity with customized small panels, but tumor tissue has to be obtained first, which results in the inability to eliminate tumor heterogeneity and to detect secondary resistance mutations, while tumor-agnostic assays have the advantage of detecting mutations not detected in the tissue due to tumor heterogeneity and of detecting secondary resistance mutations, but are limited in their sensitivity. Tumor-agnostic assays have the advantage of detecting undetected mutations due to tumor heterogeneity as well as secondary drug-resistant mutations, but they are not capable of ultra-high depth sequencing and have limited sensitivity. Therefore, as mentioned above, different technologies for MRD detection have their own advantages and disadvantages, and it is not necessary to say that one strategy is better than the other, but more clinical validation is needed. What’s more, for the definition criteria of MRD positivity, most clinical studies define the presence of more than one somatic mutation (SNV, CNV, InDel, fusion, etc.) in ctDNA as MRD positivity in blood specimens (20–22). In addition, other studies have used different criteria to determine MRD positivity. The TRACERx study (23) defined MRD positivity as the presence of more than two mutations in a single blood sample. The CAPP-seq study (24), on the other hand, calculated ctDNA detection coefficients based on the Monte-Carlo statistical simulation method, which was considered positive for MRD when the coefficient was less than 0.05. This shows that the criteria for determining MRD positivity are currently still to be further explored in academia. Finally, for the specific optimal time point for MRD monitoring, most studies take about one month after radical treatment as the optimal sampling time (25), while the DYNAMIC study collected different data including preoperative (time A), 5 min after tumor resection (time B), 30 min (time C), 2h (time C), postoperative day 1 (time P1), postoperative day 3 (time P2), and postoperative month 1 (time P3). Blood samples from different time points, including 5min (time B), 30min (time C), 2h (time C), day 1 postoperatively (time P1), day 3 postoperatively (time P2), and 1 month postoperatively (time P3), were tested for the frequency of mutant alleles in peripheral blood plasma by using next-generation sequencing to explore the ctDNA half-life and the optimal time point for MRD detection in the baseline period after lung cancer surgery. The study finally concluded that ctDNA testing on day 3 after R0 resection of the tumor can be used as the baseline time for MRD monitoring after lung cancer surgery (20). Therefore, the optimal time point for MRD testing at baseline needs to be further explored. Moreover, panel design is difficult because of tumor heterogeneity, with each patient having a different solid tumor type that is unique to them and each patient carrying only a very small number of identical genetic mutations. Furthermore, a very small amount of ctDNA (<1%) is generally released into the peripheral blood in the early solid tumor stages, which means that ctDNA testing of solid tumors must have a higher detection sensitivity than MRD testing of leukemia (most of which is tumor DNA). Finally, ctDNA has a considerably lower abundance of mutations than tissue DNA and requires extremely sensitive assays.

In this study, we propose to use MRD status as a predictive marker for the efficacy of radical chemoradiotherapy and immunoadjuvant therapy in esophageal squamous cell carcinoma, to prospectively explore the MRD results based on ctDNA detection, to suggest the risk of recurrence of the patients at an earlier stage, and to evaluate the clinical practicability of the efficacy of concurrent chemoradiotherapy and adjuvant immunotherapy and its application value in patients with inoperable esophageal squamous cell carcinoma, so as to screen the patients who can really benefit significantly from the adjuvant immunotherapy, as well as to try to identify the basis for the selection of the dose of radical radiotherapy. Currently, the main targets for MRD detection include: circulating tumor DNA (ctDNA) or circulating tumor cells (CTC), which are circulating tumor cells present in the peripheral blood spontaneously or for medical reasons, and whose detection consists of a two-step process of isolation and enrichment of the cells and analytical assay; ctDNA, a kind of free DNA in the blood, originates from either CTCs or from necrosis and apoptosis of tumor cells released from metastatic foci; and ctDNA, a kind of DNA in the blood, originates from either CTCs or tumor cells released from necrotic and apoptotic foci. released by necrotic and apoptotic cells in CTCs or metastatic foci. Compared with the MRD test of ctDNA, the technical operation of CTC in MRD test is more convenient, lower cost and easier to be accepted by patients, and there are already several NMPA-approved CTC test products in China. Compared with MRD testing of CTC, ctDNA testing is more sensitive, can predict acquired drug resistance, and is more widely used, and has been recommended by several guidelines and expert consensus. In addition, the latest research shows that the combination of CTC and ctDNA can more accurately monitor the presence of MRD in solid tumors after surgery and achieve accurate recurrence prediction (26, 27), and the combination of CTC and ctDNA opens up a new era of accurate MRD monitoring.

In conclusion, the ECMRD-001 study firstly offers a platform for assessing changes in the MRD status of patients with inoperable stage II–III ESCC before and after radical CCRT combined with immunotherapy after neoadjuvant immunochemotherapy, followed by adjuvant immunotherapy. If this study can demonstrate significant differences in treatment efficacy and tumor recurrence in patients with inoperable stage II–III ESCC, ctDNA-based MRD analysis, and ctDNA-based MRD-guided diagnosis and treatment should be implemented clinically to improve treatment efficacy and reduce tumor recurrence in such patients.

## Ethics statement

The studies involving humans were approved by the Ethics Committee of the Fourth Hospital of Hebei Medical University. The studies were conducted in accordance with the local legislation and institutional requirements. The participants provided their written informed consent to participate in this study. Written informed consent was obtained from the individual(s) for the publication of any potentially identifiable images or data included in this article.

## Author contributions

HW: Conceptualization, Data curation, Formal Analysis, Investigation, Methodology, Software, Supervision, Validation, Visualization, Writing – original draft, Writing – review & editing. XYZ: Writing – review & editing. XHZ: Writing – review & editing. CS: Writing – review & editing. WD: Writing – review & editing. WS: Funding acquisition, Project administration, Resources, Supervision, Writing – review & editing.
