# Supplementary material for: Minimal residual disease guided radical chemoradiotherapy combined with immunotherapy after neoadjuvant immunochemotherapy followed by adjuvant immunotherapy for esophageal squamous cell cancer (ECMRD-001): a study protocol for a prospective cohort study
Source: Front Immunol. 2024 Jan 11;14:1330928. doi: 10.3389/fimmu.2023.1330928 (PMC10808458; doi:10.3389/fimmu.2023.1330928)
Supplement: Supplementary file 1 [file DataSheet_1.doc]

**Supplementary Material 1. Sample size calculation**

This study used PASS software (version 15.05, NCSS, LLC. Kaysville, Utah, USA) to estimate the sample size. Based on our study’s aim, we set the parameter Alpha (two sides) to 0.05, the parameter power to 0.80, the parameter Ta (duration of enrolment) to 1 year, the parameter Tf (follow-up time) to 3 years. According to RTOG 85-01(1) and RTOG 94-05(2) studies, we set the parameter n0 (3-year OS rate after radical chemoradiotherapy for inoperable esophageal cancer based on NCCN guidelines as a control) to 0.3, the parameter n1 (expected 3-year OS rate) to 0.5, the parameter M0 (median OS of the control group mentioned above) to 1.51 years, expected HR to 0.8, the parameter k to te system default value 1. Based on the calculations, a sample of 46 cases was needed to complete this study. Considering the possibility of sample shedding during the course of the study, 56 samples were finally used as the sample size for this study.


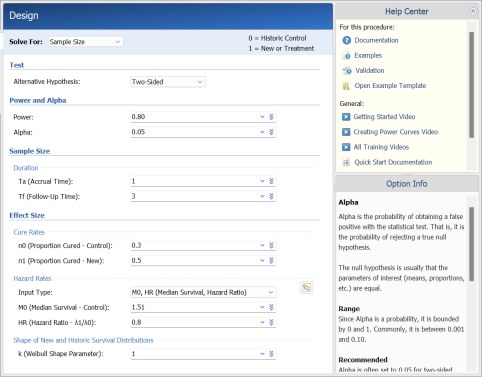


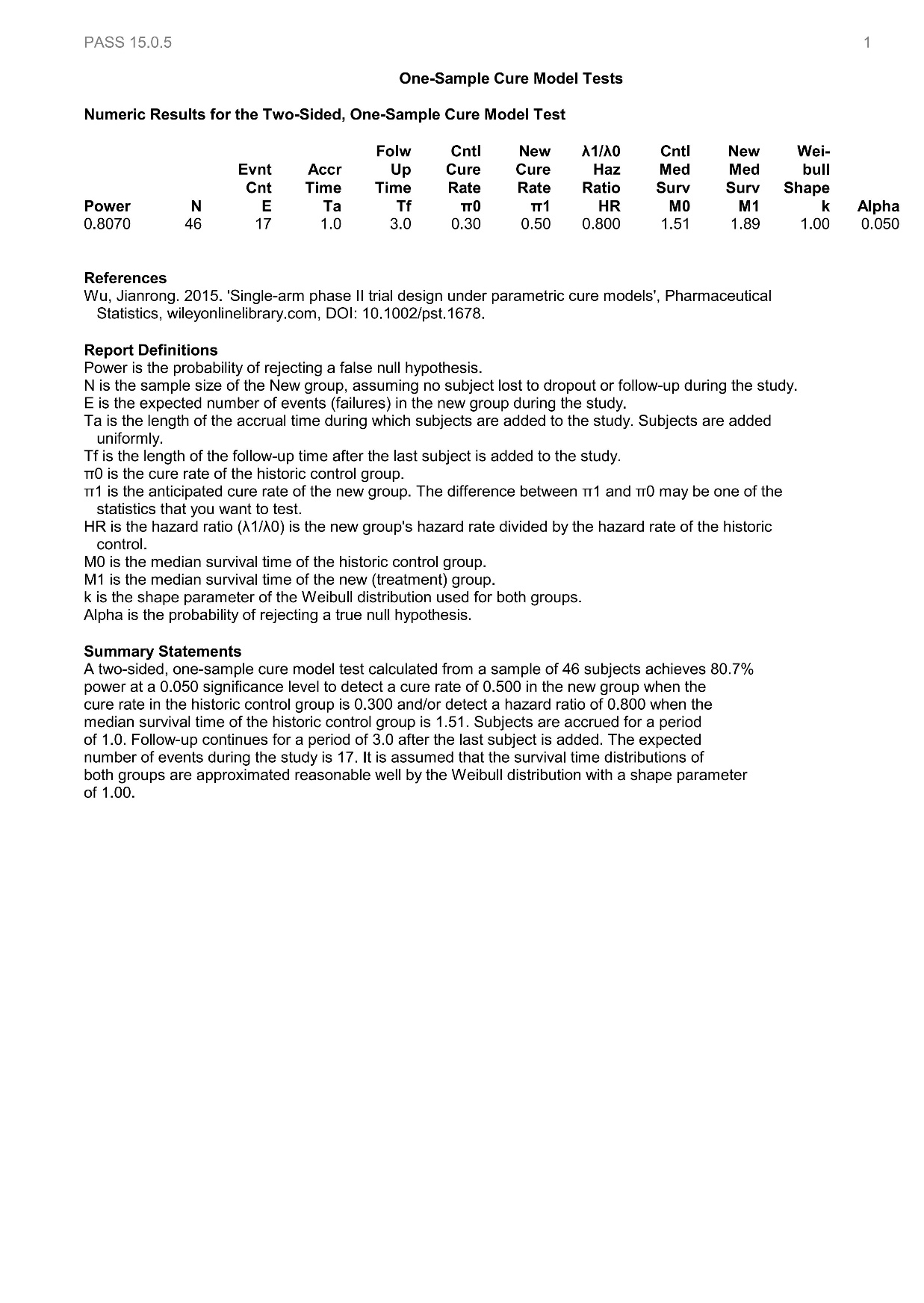


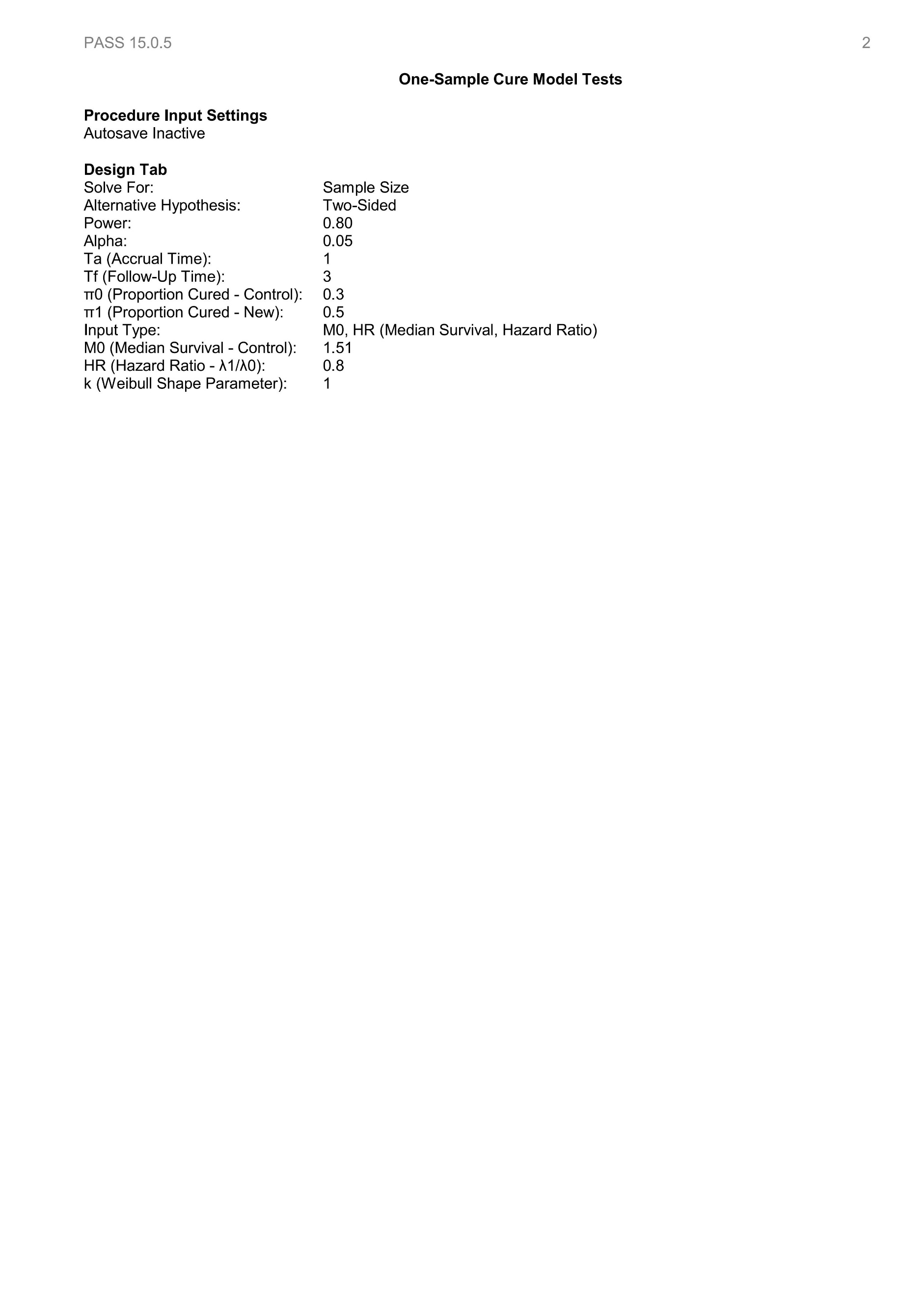


**References**

1. Cooper JS, Guo MD, Herskovic A, Macdonald JS, Martenson JA, Jr., Al-Sarraf M, et al. Chemoradiotherapy of locally advanced esophageal cancer: long-term follow-up of a prospective randomized trial (RTOG 85-01). Radiation Therapy Oncology Group. Jama. 1999;281(17):1623-7.

2. Kachnic LA, Winter K, Wasserman T, Kelsen D, Ginsberg R, Pisansky TM, et al. Longitudinal Quality-of-Life Analysis of RTOG 94-05 (Int 0123):A Phase III Trial of Definitive Chemoradiotherapy for Esophageal Cancer. Gastrointest Cancer Res. 2011;4(2):45-52.
